# Supplementary material for: Effectiveness and Safety of Ayurvedic Medicines in Type 2 Diabetes Mellitus Management: A Systematic Review and Meta-Analysis
Source: Front Pharmacol. 2022 Jun 8;13:821810. doi: 10.3389/fphar.2022.821810 (PMC9213670; doi:10.3389/fphar.2022.821810)
Supplement: Supplementary file 1 [file DataSheet1.zip › Supplementary Material/Forest and Funnel Plots/Syzygium cumini/PPBG.pdf]

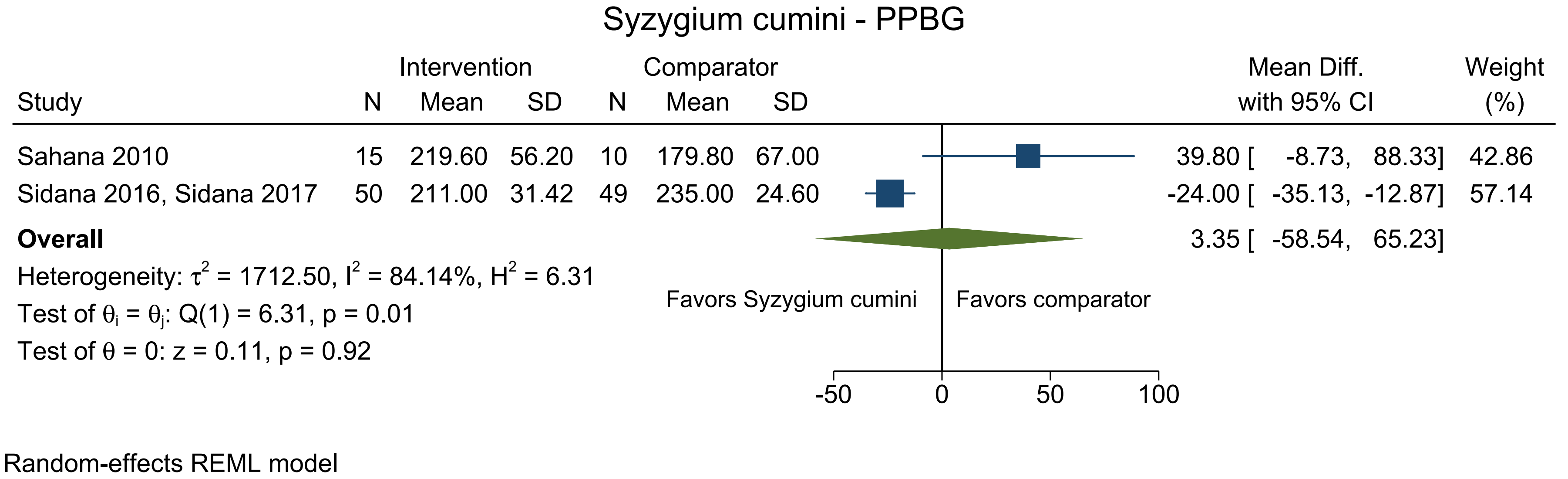
Syzygium cumini - PPBG
Intervention
Comparator
Study
N
Mean
SD
N
Mean
SD
Mean Diff. with 95% CI
Weight (%)
Sahana 2010 15 219.60 56.20 10 179.80 67.00 39.80 [-8.73, 88.33] 42.86
Sidana 2016, Sidana 2017 50 211.00 31.42 49 235.00 24.60 -24.00 [-35.13, -12.87] 57.14
Overall 3.35 [-58.54, 65.23]
Heterogeneity: τ² = 1712.50, I² = 84.14%, H² = 6.31
Test of θi = θj: Q(1) = 6.31, p = 0.01
Test of θ = 0: z = 0.11, p = 0.92
Favors Syzygium cumini
Favors comparator
-50
0
50
100
Random-effects REML model
